# Pathogenesis of type 1 diabetes: lessons from natural history studies of high-risk individuals

**DOI:** 10.1111/nyas.12021

**Published:** 2013-01-29

**Authors:** Natalie Nokoff, Marian Rewers

**Affiliations:** 1 Department of Pediatrics; 2 Barbara Davis Center for Childhood Diabetes, University of Colorado School of Medicine

**Keywords:** type 1 diabetes, autoimmune, beta cell, islet autoantibody

## Abstract

Type 1 diabetes (T1D) is an autoimmune disease characterized by known genetic risk factors with T cell–mediated infiltration and destruction of the beta cells within pancreatic islets. Autoantibodies are the most significant preclinical marker of T1D, and birth cohort studies have provided important insights into the natural history of autoimmunity and T1D. While HLA remains the strongest genetic risk factor, a number of novel gene variants associated with T1D have been found through genome-wide studies, some of which have been linked to suspected environmental risk factors. Multiple environmental factors that have been suggested to play a role in the development of T1D await confirmation. Current risk-stratification models for T1D take into account genetic risk factors and autoantibodies. In the future, metabolic profiles, epigenetics, as well as environmental risk factors may be included in such models.

## Introduction

Type 1 diabetes (T1D) is characterized by autoimmune destruction of the insulin-secreting beta cells in the pancreas. It is a common condition in Europe and the United States, and its prevalence continues to rise. Its management requires lifelong adherence to therapies and frequent interactions with healthcare professionals. For these reasons, research into the natural history of T1D as well as those who are at increased risk has major implications for doctors, patients, and health systems.

This review focuses on results of cohort studies of high-risk individuals, in particular, the Diabetes Autoimmunity Study in the Young (DAISY) in the United States, the Type 1 Diabetes Prediction and Prevention Project (DIPP) in Finland, and the BABYDIAB in Germany. We discuss the current understanding of the most important predictor of T1D: the presence and number of autoantibodies in addition to genetic risk factors. Recent findings in the area of metabolomics will be covered, as data about serum metabolites may factor into risk models of T1D in the future. The review concludes with a discussion of the role of environmental risk factors and their potential interaction with genetic variants.

## Predicting type 1 diabetes

T1D affects 1.4 million people in the United States,^1^ and its incidence has doubled over the past 20 years.^2^–^4^ In the United States, the prevalence of T1D in youth under age 20 has increased by 23% from 2001 to 2009.^5^ While children are the most visibly affected, half of T1D patients are diagnosed after age 20 and the life-time risk now exceeds 1% in North America and Europe. It is thought that T1D is caused by one or more environmental factors interacting with a relatively common genetic background, but the specific cause(s) remain elusive.

Prospective cohort studies of individuals at increased risk, such as the DAISY in the United States,^6^ BABYDIAB in Germany,^7^ and the Finnish DIPP study,^8^,^9^ have established that positivity for islet autoantibodies precedes the diagnosis of T1D, usually by years. The participants in each study differ slightly. The BABYDIAB cohort consists of offspring of parents with T1D, while the DIPP screened infants in the general population, including first-degree relatives, for HLA types and stratified based on HLA risk. The DAISY cohort consists of two groups: first-degree relatives of individuals with T1D and individuals from the general population who had HLA typing of cord blood and, like the DIPP study were stratified based on HLA risk. Identification of the environmental causes of T1D requires prospective assessment of multiple exposures before and after the development of islet autoimmunity. A person with persistent islet autoimmunity may benefit from interventions to prevent diabetes and, even in absence of prevention, avoid life-threatening diabetic ketoacidosis at diagnosis. Those who do not progress to diabetes, despite long-term persistent islet autoimmunity, may help us to understand mechanisms of autoimmunity, tolerance and regeneration. T1D shares genetic determinants with common autoimmune diseases like celiac disease and autoimmune thyroid disease, as well as the less frequent rheumatoid arthritis and Addison disease. At the age of 18 years, one or more of these conditions affect at least 3% of the general population. Despite benefits of early diagnosis,^10^ there are currently no universal screening programs and the resultant delay in diagnosis leads to significant morbidity and cost. Progress in multiplexing immune assays and genotyping has set the stage for an integrated approach to screening and early treatment.

T1D is characterized by destruction of the pancreatic beta cells by T cells. Islet autoantibodies are not thought to cause direct damage to the beta cells, and in mouse models anti-islet antibodies alone do not precipitate diabetes.^11^ However, in mouse models, autoantibodies have been shown to enhance accumulation of islet-reactive CD4^+^ T cells and promote diabetes among mice who already have an increased frequency of islet-reactive CD4^+^ T cells.^11^ Yet, the detection of islet autoantibodies in serum is currently the most reliable diagnostic test for type 1a (autoimmune) diabetes in subjects with hyperglycemia. Islet autoantibodies are also useful preclinical markers for risk of developing T1D. The autoantibodies that help to define pre-T1D and T1D include: insulin autoantibody (IAA),^12^ glutamic acid decarboxylase antibody (GADA),^13^ insulinoma-associated protein 2 autoantibody (IA-2A),^14^ and zinc transporter 8 antibody (ZnT8A).^15^ Testing for at least two of these autoantibodies at diagnosis is now considered standard of care in T1D. ZnT8A, GADA, IA-2A or IAA are present at onset of T1D in more than 90% of subjects,^15^ although the rates vary by ethnicity and age. In the U.S. SEARCH for Diabetes in Youth study, 52% of newly diagnosed children were positive for GADA, 60% were positive for IA–2A, and 38% were positive for both.^16^ In contrast, the Childhood Diabetes in Finland Study Group found that 91% of children with newly diagnosed T1D were positive for at least two autoantibodies, and 71% for three or more. IA–2A was detected in 86% of cases.^17^ Among the general population, all subjects positive for both GADA and IA–2A (mean age 11.8 years) developed T1D over the next 21 years.^18^ ZnT8A are present in 60–80% of new-onset T1D patients, and in 25% of those who are negative for GADA, IA2A, IAA, and islet cell autoantibodies (ICA).^15^ ZnT8A tend to emerge later than GADA and IAA in prediabetes.^15^ Among Japanese patients with new onset T1D, ZnT8A were detected in 58% of patients with acute-onset T1D, 20% with slow onset and in none with fulminant T1D.^19^

Prospective cohort studies, such as those summarized in Table 1 have made major contributions to the understanding of the natural history of islet autoimmunity and the etiology of T1D. For instance, DAISY has made the leap from studying the pathogenesis of T1D in relatives of affected patients to large-scale general population newborn screening and follow-up of high-risk children without an affected relative, since fewer than 10% of T1D patients belong to this category.^20^ Among children with identical *HLA-DR,DQ* genotypes, the incidence of islet autoimmunity is dramatically higher in children with a first-degree relative with T1D, compared to the general population, pointing to the importance of environmental factors and/or non-HLA class II genes.

**Table 1:** Prospective cohort studies of T1D natural history

Year started	BABYDIAB Germany 1989	DAISY Colorado 1993	DIPP Finland 1994	TEDDY Four countries 2004
First-degree relatives (*n*)	1650 offspring	1,120 offspring siblings	8,150	923
General population (*n*)	–	1,422		7,754
Persistent islet Ab+ (*n*)	149	183	537	450^a^
Diabetes (*n*)	47	71	320	126^b^

a As of October 2012, 800 cases expected by 15 years of follow-up.

b As of October 2012, 400 cases expected by 15 years of follow-up.

NOTE: The BABYDIAB consists of offspring of parents with T1D; DAISY has two groups: first-degree relatives of T1D and high-risk individuals from the general population; DIPP screened infants in the general population, including first-degree relatives, for HLA types; finally, the TEDDY cohort consists of newborns with a first-degree relative with T1D as well as those from the general population enrolled from six clinical centers in four countries (personal communication from Ziegler, Simell, and Rewers, October 2011).

DAISY reported the first ever population-based estimates of the incidence of islet autoimmunity among children in the in general population.^6^ While islet autoantibodies were found in 3.7% of cord blood samples, they appeared to be maternal of origin and were not predictive of subsequent development of islet autoimmunity.^21^ Islet autoantibodies are thought to cross the placenta and there is a correlation between the presence of autoantibodies in the cord blood of neonates born to mothers with T1D and the levels of autoantibodies in the maternal circulation.^22^,^23^ The maternal antibodies present in the neonate typically become undetectable within the first year of life.^24^,^25^ Yet, maternal islet autoantibodies may have a protective effect. In the German BABYDIAB cohort, offspring born to mothers with T1D who were positive for GADA or IA-2A at birth (measured in cord blood) were at lower risk of multiple autoantibody positivity at five years and T1D at eight years than offspring who were autoantibody negative.^26^ This is further supported by studies that have shown that the offspring of mothers compared to fathers with T1D have a decreased risk of developing islet autoimmunity and T1D.^27^–^30^ Therefore, in contrast to mouse models in which offspring are protected from diabetes via removal of maternally transmitted immunoglobulin,^31^ at least one study in humans suggests that exposure to GADA and IA-2A *in utero* may be protective against development of islet autoimmunity and T1D. It is important to note that the protective effect of maternal GADA and IA-2A was most pronounced in children without the high-risk HLA genotype and results were not significant in children with the high-risk genotype.^26^ Therefore, genetic and possibly epigenetic factors are likely to play a role.

In the 1990s, it was shown that the number of autoantibodies present is important for predicting risk of T1D among first-degree relatives of those with T1D.^32^,^33^ DAISY demonstrated that over 70% of children expressing multiple islet autoantibodies progress to T1D in 10 years compared to 15% of those with one autoantibody. Once islet autoimmunity spreads to more than one autoantigen, the progression to T1D is only a matter of time and the rate of progression is linear and hardly influenced by the *HLA-DR,DQ* genotype or family history of T1D. Furthermore, the age of appearance of the first autoantibody and the levels of IAAs (but not GAD65 or IA-2) are major determinants of the age of onset of diabetes.^34^

The DIPP study in Finland has followed high-risk children based on HLA since 1994. At a mean follow-up of 7.7 years, approximately 20% of subjects had converted to autoantibody positivity, and about 15% of those for multiple autoantibodies.^35^ The peak incidence of seroconversion occurred in the second year of life for IAA, GADA and IA-2A, with positivity for IAA occurring first, early in the second year of life; IA–2A was never the first to appear.^35^ Children very rapidly progressed from single to multiple autoantibody positivity, while the median time from seroconversion to clinical T1D was 2.47 years (95% CI 0.18–7.13 years).^35^ Similarly, in the German BABYDIAB birth cohort of children with a parent with T1D, seroconversion was greatest at age nine months to two years and IAAs tend to occur first.^36^ These findings were confirmed in the BABYDIET cohort of children with both the high-risk HLA genotype and a first-degree relative with T1D.^36^

Normal but increasing HbA1c levels for up to two years before diagnosis foreshadows progression to T1D^37^—an important observation for potential change in diabetes diagnostic criteria that is now being confirmed using pooled data from TEDDY, TRIGR, and TrialNet. Children followed by DAISY to T1D have avoided DKA and hospitalization at diagnosis, had higher C-peptide levels and lower blood glucose, as well as lower HbA1c and insulin dose during the initial year postdiagnosis than community controls, likely due to earlier diagnosis.^10^ In a retrospective analysis of data from the Diabetes Control and Complications Trial (DCCT), higher baseline C-peptide levels were associated with lower rates of microvascular complications (retinopathy and albuminuria), regardless of whether they were (at the time) receiving intensive or standard treatment.^38^ It is unclear whether earlier diagnosis (and thus higher C-peptide levels) or a slower rate of beta cell destruction via unknown factors results in lower rates of microvascular complications.

**Figure 1:**
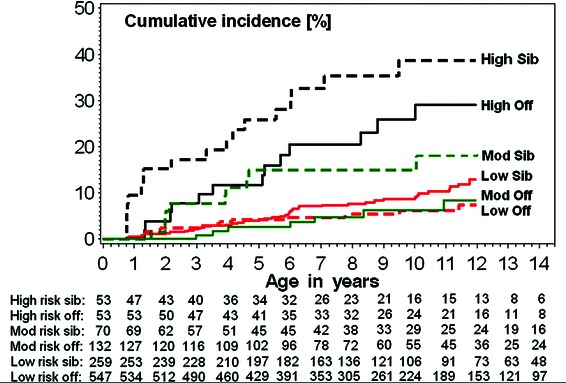
Cumulative incidence of persistent islet autoimmunity in siblings or offspring of a person with T1D. The above figure represents the cumulative incidence of persistent islet autoimmunity among siblings (sib) or offspring (off) of individuals with T1D in the DAISY cohort. The high-risk group consists of those with the genotype HLA-DR3/4,DQB1* 0302, the moderate-risk with HLA-DR3/3 or DR4/4,DQB1*0302 or DR1,DQB1*0101/4,DQB1*0302, or DR8, DQB1*0402/4, DQB1*0302, DR4,DQB1*302/DR9,DQB1*303 genotypes, and the low-risk with all other genotypes. Below the figure is the number of subjects in each group at each age.

The incidence of islet autoimmunity is much higher in relatives of T1D patients, particularly in siblings (39%, by the age 12) and offspring (29%) with the HLA-DR3/4,DQB1*0302 genotype that is represented as the “high-risk” group in Figure 1 (unpublished data, from DAISY). These groups are optimal for potential primary prevention trials. In Cox proportional hazards analyses, children with the high-risk HLA-DR3/4,DQB1*0302 genotype and a diabetic relative had a hazard ratio (HR) for islet autoimmunity of 3.8 (2.2–6.3) compared to those without a relative. Importantly, the HR estimate five years ago among those with the high-risk genotype was 10 (3.6–28), showing that, with extended follow-up of the cohort, high-risk general population children “catch up” in risk to first-degree relatives. The HR was higher in siblings (5-fold) than in offspring (2.7-fold). In contrast, among children with other HLA genotypes, having a diabetic relative increased the risk only 1.6-fold (1.1–2.3). Ethnicity and gender were not predictive when family history and HLA were taken into consideration.

## Detection of metabolic changes in T1D

Changes in lipid and amino acid profiles of blood samples collected prospectively from birth have been linked to the expression of islet autoantibodies and T1D.^39^,^40^ Metabolomics is the study of metabolites through unbiased detection and quantification of all small molecules present in a biological sample (e.g., cells, tissues, serum) and offers an additional perspective on the natural history of T1D. Recent technological advances, including ultraperformance liquid chromatography as well as mass spectrometry, have made the study of metabolomics more feasible.^41^ There are fewer human endogenous metabolites (several thousand^42^) than expressed gene variants of the ∼3 × 10^4^ human genes and proteins (5 × 10^5^ –10^6^ variants).

In the Finnish DIPP cohort of genetically susceptible neonates, metabolite profiles of 56 children who had progressed to T1D were compared to 73 nondiabetic permanently autoantibody-negative controls at different time points. Compared to controls, those who went on to develop T1D had reduced levels of succinic acid and phosphatidylcholine at birth, reduced triglycerides and antioxidant ether phospholipids throughout the follow-up period, and increased levels of proinflammatory lysophosphatidylcholines prior to seroconversion. Furthermore, those who had progressed to T1D had lower levels of ketoleucine and elevated glutamic acid before the appearance of IAA and GADA.^39^ The elevated levels of lysophosphatidylcholines are thought to be a marker for increased oxidative stress before the appearance of islet autoantibodies.^43^ Phosphatidylcholine is an important source of choline in the body and in mice it functions as an epigenetic regulator^44^ and its metabolism depends on the composition of intestinal microbiota.^45^ Studies in mouse models suggest that the intestinal microbiota interactions with the innate immune system to modify T1D progression.^46^ Further studies in humans are needed to determine the significance of these findings.

In the German BABYDIAB cohort of children of parents with T1D, the metabolite profiles of 13 children who developed autoantibodies by age two were compared with 22 children who seroconverted after age eight and to autoantibody-negative children (matched for age, date of birth, and HLA genotype). Autoantibody-positive children had elevated odd-chain triglycerides and polyunsaturated fatty acid-containing phospholipids compared to autoantibody-negative children.^40^ Odd-chain fatty acids come from the diet and are found in milk that has been linked to development of autoimmunity and T1D. Furthermore, children with early seroconversion had substantially lower levels of methionine compared to those who developed autoantibodies later as well as those who remained negative for autoantibodies. The authors point out that methionine has important roles in protein synthesis, transmethylation reactions, catabolism of choline, and amino acid metabolism as well immune system functions and DNA methylation.^47^ Contradictory to the DIPP metabolomics study that showed lower glutamine levels prior to seroconversion,^39^ the BABYDIAB study showed elevated levels not only prior to seroconversion, but also in control children.^40^

There are important differences between the DIPP and BABYDIAB cohorts. The DIPP consists of subjects at increased genetic risk recruited from the general population, whereas BABYDIAB consists of offspring of parents with T1D. In the BABYDIAB study, metabolic data were only evaluated prior to autoantibody positivity, not before diagnosis of T1D as in DIPP. Additionally, the numbers of subjects tested were small in each study. Methionine levels were not tested in the DIPP study, although both methionine and choline are epigenetic regulators that may be important markers of epigenetic changes that could characterize progression to autoimmunity and T1D. The significant though sometimes disparate results in each study point to potential contributions of the host diet, microbiome or epigenetic changes, which may, in fact, differ between countries. These early studies illustrate that there may be early markers in the metabolome prior to seroconversion or development of T1D. As the authors point out, the specific metabolic markers may be clues to perturbations in glucose metabolism, amino acid metabolism, oxidative stress, DNA methylation, or T cell regulation. Furthermore, age, gender, diet, and the gut microbiome have all been shown to affect the metabolic phenotype^48^–^50^ and many of these factors have recently been reviewed.^51^ It remains to be seen whether these results will be replicated in other prospective cohort studies.^39^,^40^ Together with genetic risk and autoantibodies, metabolomics data may, in the future, be used in T1D risk stratification.

## Genetics

### HLA

The human leukocyte antigen (HLA) locus is located on chromosome 6p. The class I genes include *HLA-A*, *HLA-B*, *HLA-C*, and class II genes *HLA-DP*, *HLA-DQ* and *HLA-DR*. Eight of these genes are highly polymorphic and play a role in immune responses: *HLA-DPA1*, *HLA-DPB1*, *HLA-DQA1*, *HLA-DQB1*, *HLA-DRB1* on the class II loci; and, *HLA-A*, *HLA-B* and *HLA-C* on class I loci. The high-risk HLA class II genes represent the strongest genetic association with T1D, and individuals with the *HLA-DR3-DQ2/DR4-DQ8* genotype are at approximately 20-fold increased risk for T1D compared to the general population.^52^ Furthermore, the high-risk HLA class II genotype accounts for about 30–50% of the genetic risk in T1D.^52^ The high-risk genotype is present in 2.4% of all newborns.^53^ By the age of 15, 5% of children with the high-risk genotype will develop islet autoimmunity and T1D, compared with only 0.3% in the general population. Furthermore, individuals with the high-risk HLA genotype (DRB1*03,*04; DQB1*0302) and at least two family members with T1D have a 50% risk of developing T1D.^54^ Among those with the *HLA-DR3/4-DQ8* or *DR4/DR4* genotypes, children with no first-degree relatives with T1D have only a 5% risk of developing islet autoimmunity, compared to a 20% risk if they have a first-degree relative with T1D.^55^ Additional HLA class II genotypes confer moderately increased risk for T1D, while others are protective.^56^

Among HLA class I genes, one of the strongest associations with T1D has been reported for B*39,^57^ which is likely involved in antigen presentation to cytotoxic CD8^+^ lymphocytes.^58^ In the DIPP cohort, the HLA-B*39 allele was associated with progression to T1D among children with either one or two autoantibodies.^59^ *HLA-A24* has been associated with more rapid islet destruction and progression to T1D.^60^ On the other hand, the A*03 allele appears to protect against progression to T1D among children with the *HLA-DR3/DR4* genotype and the presence of one or two autoantibodies.^59^ However, the class I alleles are not currently incorporated into risk models for T1D.

### Non-HLA genes

Genome-wide association studies (GWAS) have identified over 40 non-HLA polymorphisms that are associated with T1D,^61^ and a number of novel loci have been confirmed in prospective population-based studies. However, jointly they confer only a small additional risk compared to the effect of *HLA-DR, DQ*. Rather than an exhaustive review of all non-HLA genes associated with T1D (which have been reviewed elsewhere^62^), we will focus on those that confer the greatest odds or have been linked to potential environmental risk factors.^62^

One approach to validating GWAS findings is the use of prospective cohort analysis. Recently, the association of 20 genes with development of islet autoimmunity and T1D was tested among non-Hispanic white subjects with the high-risk *HLA-DR, DQ* genotype in the DAISY cohort. Variants or polymorphisms in *UBASH3A* (a suppressor of T cell receptor signaling) and *PTPN22* predicted development of islet autoimmunity and T1D when controlling for family history and presence of the HLA-DR3/4-DQB1*0302 genotype. Polymorphisms in the *INS* gene predicted development of T1D.^63^ Although the effect of each individual gene is small, the combination of family history of T1D, the HLA-DR3/4-DQB1*0302 genotype, and the susceptibility variants of *PTPN22, UBASH2,* and *INS* increased the risk of islet autoimmunity 16-fold and that of T1D 40-fold.^63^

Similarly, in the DIPP cohort of children with the high-risk HLA genotype and at least one autoantibody, the presence of the *PTPN22* 1858T allele was strongly associated with progression to T1D.^64^ However, the *INS*-23 HphI AA genotype was not associated with progression to T1D. The authors hypothesized that the high-risk *INS* genotype is likely involved in the induction and early phases of beta cell autoimmunity and the high-risk *PTPN22* in the later stages.^64^

Of the non-HLA genes, the protein tyrosine phosphatase nonreceptor type 22 (*PTPN22)* gene located on chromosome 1p13 has the strongest association with T1D.^65^ *PTPN22* codes for a lymphoid-specific phosphatase that is expressed in lymphocytes and is an inhibitor of T cell activation.^65^ Substitution of arginine for tryptophan at position 620 disrupts binding between PTPN22 and the intracellular kinase, Csk, altering responsiveness of T and B cells to receptor stimulation.^66^ This leads to decreased inhibition of T cell activation, and promotes multiorgan autoimmunity. Among individuals with the high-risk HLA genotype for T1D, the *PTPN22* 1858T allele is independently associated with the development of persistent islet autoimmunity.^67^ Compared to healthy controls, individuals with T1D are more likely to have either one or two copies of 1858T allele.^66^ In a recent meta-analysis of *PTPN22*, the 1858T allele was significantly associated with T1D across different ethnic groups (odds ratio 1.9, 95% CI 1.859–2.041).^68^ *PTPN22* is also associated with rheumatoid arthritis, systemic lupus erythematosus, Graves’ disease, and Crohn disease.^66^

Insulin has been shown to be an important, if not the most important, antigen in both humans and in mouse models of T1D. The insulin gene (*INS*) on chromosome 11 encodes for preproinsulin, which is converted to proinsulin, and finally to insulin after removal of the C-peptide. The *INS* gene is transcribed and translated in the thymus. Upstream of the *INS* promoter region lies the *IDDM2* locus, a polymorphism that consists of a variable number of tandem repeats (VNTR) of a consensus sequence at the 5′ coding region and is one of the strongest risk factors for T1D aside from HLA.^69^ Alleles of proinsulin at this locus are classified by the number of repeats, with class I with the fewest repeats (26–63), class II with 63–140, and class III with 141–209.^70^,^71^ Individuals who are homozygous for the class I allele have lower levels of proinsulin gene expression in the thymus,^72^,^73^ lower levels of IL-10 secretion,^74^ and higher titers of antiinsulin antibodies.^75^ There are other known polymorphisms with tight linkage disequilibrium with the *INS-VNTR* alleles, including the -23HphI and +1140A/C that have been shown to be associated with T1D.^76^ The *INS* -23 HphI A allele corresponds to the *VNTR* class I and the -23 HphI T allele to the VNTR class III.^77^ Homozygosity for the class I allele is associated with increased risk of T1D, while the class III allele is protective against T1D.^72^,^73^,^78^ There is no association between the VNTR genotype and the high risk HLA genotype.^77^,^79^ It is thought that lower levels of proinsulin gene expression in the thymus associated with the class I allele may be one mechanism by which tolerance to beta cell autoantigens is lost, and insulin has been implicated as the main autoantigen in T1D.^80^ Furthermore, an interaction between early exposure to cow's milk formula, the *PTPN22* 1858T and *INS* -23 Hph AA genotypes promoting the development of islet autoantibodies and T1D has been reported.^81^

The *IFIH1* (interferon induced with helicase C domain 1, also known as MDA5, or melanoma differentiation-associated gene 5) linkage disequilibrium block on chromosome 2q has also been found to be associated with T1D in GWAS and increased gene expression is associated with risk of T1D.^82^ IFIH1 is a cytoplasmic helicase that plays a role in detection of intracellular viral dsRNA of picornaviruses, a family of viruses that includes enteroviruses.^83^ Detection of intracellular viral RNA leads to IFIH1-activation of interferon pathways.^84^ It is hypothesized that infection with enteroviruses results in *IFIH1* activation in the pancreatic beta cell, elevated levels of interferon and enhanced expression of surface MHC-I molecules, which bind to cytotoxic CD8^+^ T cells, and result in beta cell death.^85^ Furthermore, *IFIH1* variants that result in reduced function of the IFIH1 protein are protective against T1D.^86^ The potential link between enterovirus and T1D has been studied extensively and the rate of progression from autoimmunity to T1D is significantly higher after enterovirus detection.^87^,^88^

A recent study used available GWAS data along with protein–protein interactions and identified 17 biological networks of relevance to T1D.^61^ Three of the networks contained genes involved in cytokine regulation, and the expression of novel candidate genes were confirmed in insulin-secreting beta cells.^61^ Such studies may prove to be important in the identification of novel therapies for T1D and translational research. Furthermore, a worldwide GWAS study showed that the quantity and diversity of pathogen exposure is a strong selective pressure in human evolution. Many of the genes shown to be under positive selection using this method are also known to be correlated with autoimmunity.^89^ This suggests that the genes responsible for risk for autoimmunity may have been adaptive under other environmental circumstances.

Lastly, epigenetics is an emerging area of research in T1D and may help provide important clues regarding environmental risk factors. DNA methylation and histone posttranslational modification are the two main epigenetic modifications. In autoimmune diseases other than T1D, epigenetic changes in a variety of cell types have been more extensively studied.^90^ Hypomethylation of DNA in target tissues of patients with rheumatoid arthritis, ulcerative colitis and psoriasis has been demonstrated,^91^–^93^ as well as changes in methylation patterns of CD4^+^ T cells and peripheral blood mononuclear cells in a variety of autoimmune diseases^93^,^94^ and histone acetylation in systemic lupus erythematosus.^95^ In T1D, changes in the methylation patterns of the *INS* gene promoter and CD14^+^ monocytes have been studied,^96^,^97^ as well as histone posttranslational modifications.^98^ One study mapped histone posttranslational modifications in 41 T1D susceptible regions in subjects with T1D versus normal controls. Compared to control subjects, those with T1D had variations in histone H3K9Ac at the promoter region of *HLA-DRB1* and *HLA-DQB1* genes in monocytes, which are known to be associated with T1D.^98^

Certain epigenetic studies compare those with T1D to healthy controls, whereas others examine monozygotic twins who are discordant for T1D to minimize genetic variation. A recent epigenome-wide association study identified methylation variable positions associated with T1D among monozygotic twin pairs discordant for T1D.^97^ However, they also showed that these same methylation variable positions are present among nontwin subjects with T1D both before and at disease diagnosis as well as in subjects with diabetes-associated autoantibodies who are disease-free at 12 years of follow-up.^97^ Therefore, it is unclear if significant epigenetic modifications are the cause or consequence of disease (or autoimmunity) and studies to establish the temporal origins of epigenetic changes may shed light on the timing of environmental risk factors. For example, if epigenetic variations are seen at birth, that would suggest that *in utero* factors are likely to play a role in the development of T1D; whereas, if epigenetic changes emerge later in life, this might point to different environmental risk factors. This will be important information to refine the search for environmental factors and their association with emergence of autoimmunity and T1D in large prospective studies. Already, some studies are showing associations between genetic polymorphisms known to be associated with T1D and specific environmental risk factors.

## Environmental factors

Rising incidence^99^ and outbreaks^100^ and a seasonal pattern of incidence^101^ point to environmental factors that play a role in the pathogenesis of T1D. The overall concordance rate for T1D among monozygotic twins is only about 10–40%,^102^,^103^ which shows that nongenetic factors contribute to the risk of T1D. However, T1D cohort studies have not followed twin subjects over their entire lifetime; therefore, the true concordance rate may be much higher. Even if concordance were 100%, the environment could influence timing of disease. Although, the younger the proband is diagnosed with T1D, the higher the concordance rate among monozygotic twins. If the proband is diagnosed under age five years, the concordance rate is 65%,^103^ whereas if the proband is diagnosed after age 24 years, the concordance rate is only 6%.^104^ This not only highlights the important role of genetics in the development of T1D, but also environmental or other factors given concordance rates less than 100%, particularly with diagnosis at an older age.

Furthermore, the incidence of T1D is increasing in younger children and those with lower-risk HLA genotypes.^105^,^106^ In Australia, the proportion of those with the *HLA DR3, 4* genotype decreased from 79% in 1950–1969 to only 28% in 2000–2005, while the proportion of those with intermediate-risk genotypes increased from 20% to 48% over that same time period.^107^ Furthermore, the incidence of T1D among those with the highest risk-HLA genotype remained unchanged. It is likely that one or more environmental factors are contributing to the increasing incidence despite unchanged genetic risk. There are many potential candidates, including dietary factors (such as gluten and cow's milk), obesity, viruses (particularly enterovirus), and the intestinal microbiota. We will briefly review a few key factors with a focus on pertinent results from birth cohort studies as well as factors that have been linked to genetic risk.

### Dietary factors

Several large prospective studies have made important inroads into our understanding of the role of infectious and dietary agents in triggering islet autoimmunity leading to T1D.^34^, ^53^, ^108^,^109^ A recent review of dietary factors showed that early introduction of cereals, lower intake of omega-3 fatty acids and lower maternal consumption of vegetables and potatoes are associated with increased risk of early-childhood islet autoimmunity.^110^ In the DAISY cohort, exposure to cereals before three months or after seven months of age was associated with increased risk of development of islet autoimmunity compared to those exposed between four to six months, although the association was stronger among children with the HLA-DRB1*03/04,DQB8 genotype, but did not differ based on family history of T1D.^111^ However, exposure to both gluten and nongluten cereals was associated with increased risk of islet autoimmunity. Likewise, in the BABYDIAB cohort, introduction of gluten before age three months was associated with increased risk of islet autoimmunity, but there was no association if gluten was introduced after six months of age.^108^ However, this represented only 4 out of 17 children who had exposure to gluten-containing foods before age three months and all had the high-risk HLA genotype, DRB1*03/04,DQB1*0302. In the BABYDIET study, a primary prevention trial wherein children with a first-degree relative with T1D were randomized to either early or late (6 vs. 12 months) introduction of gluten-containing cereals, delayed exposure to gluten was not associated with a decrease in the risk of islet autoimmunity.^112^ There is some evidence from the celiac literature that introduction of gluten while breast feeding reduces the risk of celiac disease.^113^,^114^ It is unclear if this could also be the case for T1D, which shares some of the genetic risk with celiac disease.

The relationship between breastfeeding or intake of cow's milk and development of islet autoantibodies and T1D in genetically at-risk infants has been a subject of controversy.^108^, ^111^,^115^ The Trial to Reduce IDDM in the Genetically at Risk (TRIGR) study is a large, prospective, multicenter, double-blind placebo controlled trial in which infants with the high-risk HLA genotype and a first-degree relative with T1D were randomized to receive either a casein hydrosylate formula or a conventional cow's milk formula.^116^ Interim analyses have shown a significant decrease in the cumulative incidence of autoantibodies among infants who received hydrolyzed formula.^117^,^118^ In the Finnish Dietary Intervention Trial for the Prevention of Type 1 Diabetes (FINDIA), 1,113 infants were randomized to receive either standard cow's milk formula, a whey-based hydrolyzed formula or a whey-based formula free of bovine insulin during the first six months of life (when breast milk was not available).^119^ Infants who received formula free of bovine insulin were significantly less likely to have autoantibodies at age three years than those who received regular cow's milk. Prior studies have shown that infants exposed to cow's milk formula before age three months had higher IgG-antibodies that bound to bovine insulin than those who were exclusively breast fed.^120^ Human insulin and bovine insulin differ by only three amino acids (two in the A-chain and one in the B-chain)^121^,^122^ and antibodies that bind to bovine insulin can crossreact with human insulin.^120^ In the DIPP study, the effect of polymorphisms that have been shown to be associated with T1D (including *INS* -23A/T, *PTPN22* 1858C/T and *CTLA-4* +49A/G) on the emergence of islet autoimmunity was studied in children who were either exposed to cow's milk based formula before or after age six months.^81^ Both the *PTPN22* and *INS* polymorphisms were associated with appearance of T1D-associated autoantibodies (ICA, IAA, GADA, IA-2A) in children exposed to cow's milk formula before age six months. These findings may help explain prior contradictory findings as genetic risk coupled with timing of certain dietary exposures seems to affect development of autoantibodies. The authors hypothesize that the *INS* gene polymorphism associated with cow's milk exposure is due to early bovine insulin exposure and impaired down-regulation of insulin-specific immunity.^81^ The *PTPN22* 1858T polymorphism affected the levels of antibodies bound to dietary insulin when bovine insulin was introduced early in life and is also thought to interact with mechanisms of tolerance in the gut. It hypothesized that there is an interaction between the intestinal microbiome, gut permeability and the development of mucosal immunity^123^ and has recently been reviewed.^51^

### Accelerator hypothesis

The accelerator hypothesis proposes that the rise in T1D (as well as type 2 diabetes) is related to increasing rates of childhood obesity and insulin resistance.^124^ There have been many studies of growth in infancy and childhood in individuals with T1D as well as in relatives at increased genetic risk. In the Australian Baby Diab study weight and body mass index (BMI) *z*-scores were found to predict development of islet autoimmunity.^125^ In the Melbourne Prediabetes Family Study, the number of islet autoantibodies, age and first-phase insulin response (FPIR, a measure of insulin secretion) predicted progression to T1D.^126^ The Diabetes Prevention Trial-Type 1 (DPT-1) found that among antibody-positive first-degree relatives of individuals with T1D, insulin resistance as measured by the homeostasis model of assessment–insulin resistance (HOMA-IR) predicted progression to T1D.^127^ Similarly, in the DiMe study in Finland, FPIR and HOMA-IR to FPIR ratio predicted progression to T1D among autoantibody-positive subjects.^128^ In the European Nicotinamide Diabetes Intervention Trial (ENDIT) trial, FPIR, number of antibodies in addition to islet-cell antibodies and 120 minute oral glucose tolerance test predicted risk of progression to T1D among first-degree relatives of individuals with T1D. Insulin resistance, as measured by HOMA-IR predicted acceleration to T1D only among those with low insulin secretion (FPIR).^129^ In the DAISY study, greater height growth velocity (but not weight or BMI growth velocity) predicted development of islet autoantibodies (hazard ratio 1.6, 95% CI 1.3–2.1) and T1D (hazard ratio 3.3, 95% CI 1.7–6.4) for a one standard deviation difference in velocity.^130^ However, in the BABYDIAB cohort, islet autoantibody-positive children were neither insulin resistant nor had an increased BMI.^131^ Results have been inconsistent between studies and further prospective studies are needed (see Table 2).

**Table 2:** Summary of previous prospective studies of the effect of body weight or insulin resistance on development of islet autoimmunity (IA) or progression from IA to T1D^132^

Study	*N*	Population	Age period (median/mean follow-up)	No. who developed IA	Predictor of IA HR (95% CI)	No. who developed T1D	Predictor HR (95% CI)	Adjusted for
Melbourne Prediabetes Family Study^126^	104	FDRs	9–39 years (4.0 years)	Not studied	Not studied	43	HOMA-IR:1.65 (1.21–2.25) Fasting insulin: 1.14 (1.06–1.22)	Age, FPIR
Childhood Diabetes in Finland Study (DiMe^128^)	77	siblings of T1D children	0.8–19.7 years (15.0 years)	Not studied	Not studied	38	FPIR (low vs. normal): 4.7 (1.9–11.6) HOMA-IR (unadjusted): 1.0 (0.84–1.3)	Age, HLA, islet autoantibodies
DPT-1^127^	356 (186 moderate risk, 170 high risk)	FDRs	6–23 years (4.3 years moderate risk; 3.7 year high risk)	Not studied	Not studied	53 in moderate risk 70 in high risk	Moderate risk: HOMA-IR: 2.70 (1.45–5.06) FPIR:HOMA-IR: 0.32 (0.18–0.57) High risk: HOMA-IR: 1.83 (1.19–2.82) FPIR:HOMA-IR: 0.56 (0.40–0.78)	Age, FPIR, A1c, islet antibodies
ENDIT^129^	213	FDRs	<25 years (4.2 years)	Not studied	Not studied	130	HOMA-IR: 1.27 (0.91–2.00)	Autoantibodies FPIR, and 2-h glucose
Diabetes Autoimmunity Study in the Young (DAISY^130^)	1714 345 had HLA-DR3/4, DQB1*0302	829 FDRs 885 GP	2–11.5 years	75	Weight: 0.61 (0.39–0.98) Weight Δ velocity: 0.88 (0.69–1.11) BMI: 0.99 (0.80–1.21) BMI Δ velocity: 0.88 (0.64–1.21) Height Δ velocity: 1.63 (1.31–2.05)	21	Weight: 0.88 (0.33–2.32) Weight Δ velocity: 1.01 (0.58–1.77) BMI: 1.12 (0.70–1.81) BMI Δ velocity: 1.28 (0.79–2.08) Height Δ velocity: 3.34 (1.73–6.42)	HLA and FDR status
BABYDIAB^131^	1650	FDRs	2–17 years	135	BMI-SDS, *n* = 1650 No difference between BMI-SDS in IA+ and IA- children HOMA-IR, *n* = 777 No difference in HOMA-IR between IA+ and IA- children	47	No difference in the time to progression to T1D by tertiles of BMI-STD at seroconversion (A. G. Ziegler, personal communication)	
Australian Baby Diab^125^	548	FDRs	0–10 years (5.7 years)	46	Birth weight-*z*: 0.86 (0.66–1.14) Weight-*z* at 2 years: 1.43 (1.07–1.93) Weight-*z* at 4 years: 1.35 (0.99–1.84)	Not studied	Not studied	Birth weight and HLA

BMI, body mass index; CI, confidence interval; FDR, first-degree relative; FPIR, first-phase insulin response; GP, general population; HLA, human leukocyte antigen; HOMA-IR, homeostasis model of assessment-insulin resistance; HR, hazard ratio; IA, islet autoimmunity; SDS, standard deviation score; T1D, diabetes. Adapted with permission.

A large multicenter consortium, the Environmental Determinants of Diabetes in the Young (TEDDY) is under way to identify environmental factors predisposing to, or protective against, islet autoimmunity and T1D.^109^ The consortium has screened 424,788 newborns for high-risk *HLA-DR* and -*DQ* genotypes in Finland, Sweden, Germany, and the United States. Of those, 8,677 were enrolled into a long-term follow-up for development of islet autoantibodies and T1D. Participating children completed the initial study visit by four months of age. They are now being followed for development of the study endpoints with a meticulous assessment of environmental exposures and a clinic visit every three months for the first four years of life. Beginning at age four, children who have been persistently autoantibody positive will continue to be followed every three months; all others are followed every six months until age 15. Parents fill out questionnaires at regular intervals and record in the “TEDDY Book” events regarding diet, allergies, vaccinations, dietary supplements, illnesses, medication, daycare, school, social groups, and significant life events. The study staff complete additional questionnaires and anthropometric measurements at each visit. Blood, stool, nasal swab, saliva, urine, toenail clippings, and drinking water are collected at different intervals. Physical activity is measured by an accelerometer starting at age five. As of October 2012, persistent confirmed islet autoantibodies have developed in 450 children and 126 subjects have developed T1D. The overarching goal of TEDDY is identification of environmental triggers of T1D that could be targeted in primary prevention trials.

## Conclusion

T1D is a polygenic autoimmune disease with incompletely elucidated environmental triggers. While a number of candidate gene variants have been identified, the HLA region explains most of the familial clustering of T1D. Non-HLA gene variants individually confer only a small risk of T1D; however, functional studies have uncovered important roles of these genes in development and progression of autoimmunity. There may be gene–environment interactions between cow's milk and *PTPN22* and *INS* or *IFIH1* and enterovirus. Additional confirmatory studies are needed as well as further investigations into possible mechanisms. The relatively new field of metabolomics may provide an important additional model for risk stratification as well as a link between environmental risk factors, intestinal microbiota, and epigenetic changes with serum metabolite markers. While these new areas of research provide exciting possibilities, early studies often reveal conflicting results. Therefore, large prospective studies of high-risk children are needed to gain insight into the environmental triggers of human T1D. This robust area of research should lead to a better understanding of the mechanisms of autoimmunity and may allow for effective preventive therapies.
